# Using Pupillometry in Virtual Reality as a Tool for Speech-in-Noise Research

**DOI:** 10.1097/AUD.0000000000001692

**Published:** 2025-07-02

**Authors:** Hidde Pielage, Bethany Plain, Sjors van de Ven, Gabrielle H. Saunders, Niek J. Versfeld, Sophia E. Kramer, Adriana A. Zekveld

**Affiliations:** 1 Amsterdam Universitair Medisch Centrum Location Vrije Universiteit Amsterdam, Otolaryngology-Head and Neck Surgery, Section Ear & Hearing, De Boelelaan 1117, Amsterdam 1081 HV, the Netherlands; 2 Eriksholm Research Centre, Rørtangvej 20, Snekkersten 3070, Denmark; 3 Manchester Centre for Audiology and Deafness, University of Manchester, Manchester, UK.

**Keywords:** Copresence, Listening effort, Pupillometry, Speech-in-noise, Virtual reality

## Abstract

**Objectives:**

Virtual reality (VR) could be used in speech perception research to reduce the gap between the laboratory and real life. However, the suitability of using VR head-mounted displays (HMDs) warrants investigation, especially when pupillometric measurements are required. The present study aimed to assess if pupil measurements taken within an HMD would be sensitive to changes in listening effort related to a speech perception task. Task load of a VR speech-in-noise task was manipulated while pupil size was recorded within an HMD. The present study also assessed if VR could be used to simulate the copresence of other persons during listening, which is often an important aspect of real-life listening. To this end, participants completed the speech-in-noise task both in the copresence of virtual persons (agents) and while the virtual persons were replaced with visual distractors.

**Design:**

Thirty-three normal-hearing participants were provided with a VR-HMD and completed a speech-in-noise task in a virtual environment while their pupil size was measured. Participants were simultaneously presented with two sentences—one to each ear—which were masked by stationary noise that was 3 dB louder (−3 dB signal to noise ratio) than the sentences. Task load was manipulated by having participants attend to and repeat either one sentence or both sentences. Participants did the task both while accompanied by two virtual agents who provided positive (head nodding) and negative (head shaking) feedback on some trials, or in the presence of two visual distractors that did not provide feedback (control condition). We assessed the effect of task load and copresence on performance, measures of pupil size (baseline pupil size and peak pupil dilation), and several subjective ratings. Participants also completed two questionnaires related to their experience of the virtual environment.

**Results:**

Task load significantly affected baseline pupil size, peak pupil dilation, and subjective ratings of effort, task difficulty, and performance. However, the manipulation of virtual copresence did not affect any of the outcome measures. The effect of task load on performance could not be assessed, as single-sentence conditions often resulted in a ceiling score (100% correct). An exploratory analysis provided some indication that trials following positive feedback from the agents (as compared to no feedback) showed increased baseline pupil sizes. Scores on the questionnaires indicated that participants were not highly immersed in the virtual environment, possibly explaining why they were largely unaffected by the virtual copresence manipulation.

**Conclusions:**

The finding that baseline pupil size and peak pupil dilation were sensitive to the manipulation of task load suggests that HMD pupillometry is sensitive to changes in arousal and effort. This supports the idea that VR-HMDs can be successfully combined with speech perception research using pupillometry. The lack of an effect of the virtual copresence manipulation on the physiological and subjective measures suggests that more advanced simulations may be required in a VR setting to study the effects of copresence. Weak evidence was found that positive feedback to participants was associated with increased baseline pupil size on subsequent trials; future studies should further examine the impact of feedback on listening.

## INTRODUCTION

Recently, there has been a call for hearing research to use tests that more closely resemble real life because findings from such studies would better reflect “real-life hearing-related function, activity or participation” (Keidser et al. 2020, p. 7S). However, imitating real-life situations is difficult to achieve within the constraints of a controlled experiment (Kvavilashvili & Ellis 2004). One solution could be to use virtual reality (VR) technology, which can create realistic virtual environments with high degrees of experimental control (Parsons 2015; Yaremych & Persky 2019; Kothgassner & Felnhofer 2020). While recent studies have started to adopt VR to increase the ecological validity of hearing research (Hohmann et al. 2020), the effect of the implementation aspects of VR on speech perception research warrants further investigation, especially when combined with pupillometry, a commonly used index of listening effort.

### Listening Effort and Pupillometry

To gain deeper insights into the cognitive processes involved in listening, researchers have increasingly focused on the concept of listening effort, defined as “the deliberate allocation of mental resources to overcome obstacles in goal pursuit when carrying out a [listening] task” (Pichora-Fuller et al. 2016, p. 5). When listening situations are adverse (e.g., because of background noise or hearing loss), part of the auditory signal is lost. The allocation of additional mental resources could aid an individual in overcoming the posed difficulties by completing parts of the missing information (Zekveld et al. 2010). While this can benefit listening performance, listening also becomes more effortful. Over time high and/or sustained listening effort could lead to fatigue (Pichora-Fuller et al. 2016), which is a common complaint of persons with hearing difficulties (Nachtegaal et al. 2009). Understanding listening efforts could thus aid us in better understanding the consequences of hearing difficulties.

Listening effort has been assessed using a variety of measures, such as electroencephalography, electrocardiography, and by means of subjective ratings (asking participants how much effort they exerted) (Strand et al. 2018). However, pupillometry has emerged as one of the most widely used methods (Zekveld et al. 2018). When individuals engage in listening tasks, especially those requiring significant mental resources, their pupils dilate. This dilation is thought to correspond with the brain allocating more resources to process the auditory information (Pichora-Fuller et al. 2016; Ohlenforst et al. 2017). Pupillometry has proven valuable because the pupil’s response to auditory stimuli can provide physiological (thus objective) data with high temporal resolution (Richter et al. 2023).

Contadini-Wright et al. (2023) postulated that pupil response is influenced by sustained arousal, instantaneous arousal, and attention, all of which have been collated under the umbrella term “listening effort.” Often, researchers are primarily interested in pupil size changes directly following the onset of speech material. These changes suggest a rapid allocation of cognitive resources in response to the speech material. A commonly used pupil metric is peak pupil dilation (PPD) relative to baseline (measured right before the onset of the speech material; baseline pupil size [BPS]). However, it may well be that listening to speech also influences sustained arousal. While it has been difficult to establish a clear definition of arousal (Pichora-Fuller et al. 2016), here we mainly refer to tonic arousal in anticipation of a listening task, which is reflected in BPS (Ayasse & Wingfield 2020; Contadini-Wright et al. 2023).

### Pupillometry and VR

Pupil size can be measured using eye trackers mounted on a screen or desk in front of a participant. However, this is not possible when participants are wearing a head-mounted display (HMD) used for VR immersion, that covers the eyes of the participant. To capture pupil size while wearing an HMD, the eye tracker must be fitted inside the HMD. Although this technology is in use, there are currently no published VR-HMD speech perception studies that have employed pupillometry. Therefore, it is not known whether pupil measurements from such an HMD setup are sufficiently sensitive to changes in effort and arousal due to changes in auditory task demand.

Outside the domain of hearing science, pupillometry, and VR have been successfully combined. Integrated HMD eye trackers have been used in pupillometry research on emotion (Zheng et al. 2020), face identification (Liu et al. 2020), and recall (Orlosky et al. 2019). However, even outside the domain of hearing science, there is little research on the combination of HMDs and pupillometry as an index of effort (Bækgaard et al. 2019). Nonetheless, it is reassuring that we recently showed that the measurement of pupil size is not confounded by the fact that HMDs promote vergence (eye rotation toward objects) when moving gaze from a far to a near target, but not convergence (reshaping of the lens of the eye), which does occur outside of VR (Pielage et al. 2023).

### Copresence and VR

In real-life situations, listening typically occurs in the copresence of other persons. When acoustical conditions are adverse, these listening situations may induce social uncertainty and involve feelings of evaluative threat. Lab-based attempts to imitate such real-life situations have found that evaluative feedback provided by the experimenter, as well as the mere presence of another person can influence the allocation of effort and/or arousal when participants are engaged in a speech-in-noise (SiN) perception task. For instance, Zekveld et al. (2019) found that receiving feedback from experimenters during a SiN task was associated with an increased pupil dilation response during listening. Pielage et al. (2021) found that when participants performed a SiN task together (by alternating sentence repetitions), they had greater PPDs (which the authors interpreted as increased effort) compared to when they did the task alone. Last, Pielage et al. (2023) and Plain et al. (2021) found that the presence of two observers during a SiN task resulted in increased pupil dilations in the latter half of the pupil response, as well as increased blood pressure, suggesting more effort and stress respectively. Unfortunately, while they are insightful, these laboratory studies are resource-intensive, complex, and difficult to control.

Researchers in social psychology have encountered similar difficulties and have proposed VR as a possible solution (for a review, see Yaremych & Persky 2019). For example, one study successfully used social situations in VR to elicit social anxiety (Dechant et al. 2017). Similarly, VR might be a promising tool for speech perception research that assesses more realistic environments that include the copresence of other persons. However, first, it is necessary to determine whether copresence manipulations can be sufficiently simulated in VR by replicating laboratory-based studies in which copresence was manipulated.

In general, the degree to which VR settings induce the same response as real-life settings depends on several characteristics. For example, in VR research, it is important to consider place illusion (PI), which describes the feeling of “being there” in the virtual environment (VE) (also referred to as presence), as well as plausibility illusion, which describes the experience that what is happening in the VE is truly happening. High levels of PI and plausibility illusion cause immediate and subconscious reactions to the VE as if it were real, regardless of users being conscious that the environment is a digital render (Slater & Sanchez-Vives 2016). As a result, behavioral and physiological responses approximate those of similar real-life environments, making findings generalizable to real life (Kothgassner et al. 2014).

### Present Study

The present study had two purposes: (1) to assess if HMD pupillometry was a sensitive measure of effort during a SiN task, and (2) to assess whether VR can be used to simulate the copresence of other persons in such a way that it will affect performance, subjective ratings and/or the pupil response of participants during a SiN task. The present study did not aim to develop and test realistic virtual test environments for listening research, it was rather a “proof-of-concept” by trying to replicate lab-based findings in the VE. Development of realistic virtual test environments was considered premature because there is insufficient knowledge about whether pupillometry can be used in VR as a measure of listening effort, and whether copresence effects on listening effort can be adequately imitated using VR. In the future, this work can be expanded upon by employing more realistic environments.

To study if HMD pupillometry was a sensitive measure of effort during a SiN task, we had participants complete a VR dichotic SiN task in which signal to noise ratio (SNR) was manipulated. This type of task has been shown to elicit strong pupil responses (Koelewijn et al. 2014). If HMD pupillometry is a sensitive measure of effort, similar results to Koelewijn et al. (2014) should be found in the present study.

To assess whether VR can be used to simulate the copresence of other persons, participants performed half of the trials in the copresence of two virtual computer-steered persons (agents) who sometimes provided non-verbal feedback about the participants’ performance by nodding or shaking their head. If VR can adequately simulate copresence during speech perception tasks, similar results to non-VR studies (Pielage et al. 2021, 2023) should be found in the present study.

Last, BPS has been related to task load manipulation (Koelewijn et al. 2014) as well as copresence (Pielage et al. 2023). In both studies, the authors hypothesized that BPS is related to the pre-allocation of cognitive resources in anticipation of a listening task. Usually, the start of a trial is marked by a cue (e.g., the onset of noise), which informs participants that they must complete a (difficult) listening task soon. The increased BPS might reflect that participants prepare for the upcoming challenge by allocating some cognitive resources in advance. If pupillometry and VR could be successfully combined, these findings should also be replicated in the present study.

## MATERIALS AND METHODS

### Participants

Thirty-three participants (20 females) aged between 19 and 40 years (mean 26.8, SD: 5.7) were recruited to take part in the study. All participants were native Dutch speakers with normal hearing (defined as pure tone thresholds of 20 dB HL or less for all octave frequencies between 0.5 and 4 kHz in both ears). Exclusion criteria were: (a) the use of psychoactive medication, (b) a history of neurological or psychiatric diseases, (c) a history of eye-related diseases, (d) diabetes, and (e) pacemakers, as cardiovascular measures were acquired as well (data described elsewhere: (Plain et al. 2024) . In total, the visit lasted approximately 2 hr and participants received 15 euros as compensation for their time.

This study was approved by the medical ethical research committee of the Amsterdam University Medical Center, location VUmc, under reference number 2018.308.

### Sample Size Calculation

An a priori power calculation was performed by considering pupil and cardiovascular data (described in Plain et al. in press). To achieve sufficient power for the cardiovascular measure, a sample of 28 was required, which was increased to 33 to account for some expected data loss (because of difficulties capturing cardiovascular data from some participants). To check if this number was sufficient for the pupil analyses, a simulation approach was used. Specifically, we simulated PPD as an outcome measure, as PPD has been found to be sensitive to both manipulations of task load and copresence/social evaluation (Ohlenforst et al. 2017; Zekveld et al. 2019; Pielage et al. 2021). For the simulations, we assumed that the manipulation of task load would have a strong effect (estimated *β* of 0.20 mm) on PPD (Koelewijn et al. 2014) and that the virtual copresence manipulation would have a relatively weak effect (estimated *β* of 0.025 mm) on PPD (Pielage et al. 2021, 2023). In line with previous findings (Pielage et al. 2021, 2023), we did not expect an interaction between the two factors. The simulation indicated that 14 participants would be sufficient to detect both pupil changes in response to the agents and in response to the task load manipulation (80% power and alpha of 0.05), which meant that 33 inclusions would be sufficient.

### Virtual Environment

**Fig. 1.**
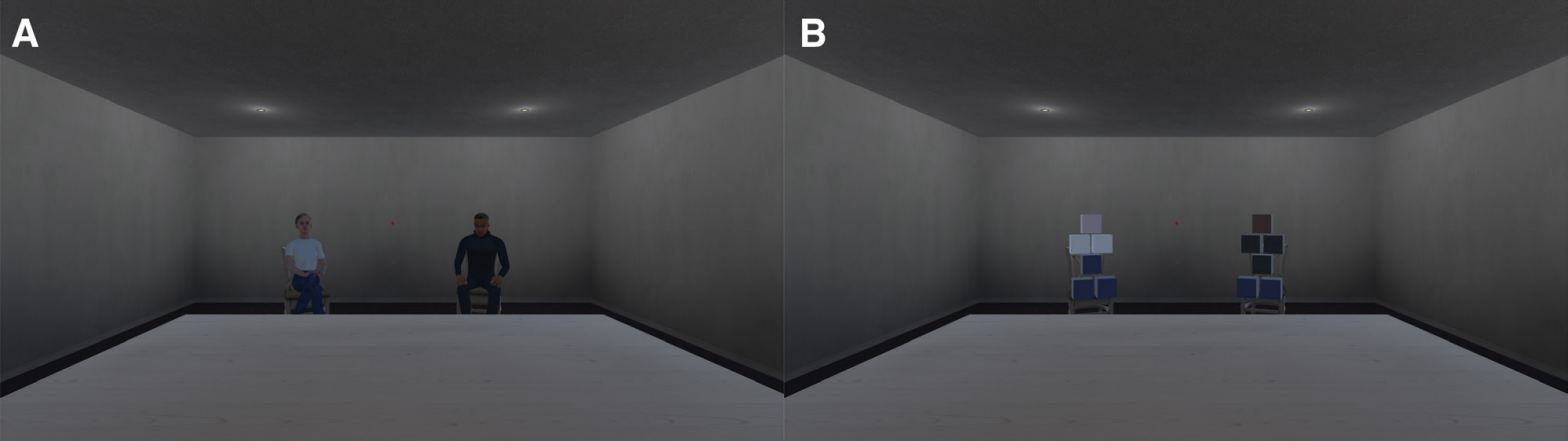
Images of the virtual environment which participants were immersed in, with either (A) the agents copresent, or (B) the agents replaced by visual distractors. While the fixation dot was present, it is barely visible in this image. In the virtual environment the fixation dot was much more salient.

During the task, participants were shown a simple VE consisting of a 9 m long by 6 m wide empty room with a desk, some chairs, and two windows. These dimensions were chosen because the perception of distance is typically underestimated in VEs as compared to the real world. Therefore, our environment can not necessarily be regarded as a one-to-one mapping to an actual room, as the distance of 9 m is likely underperceived (Adams et al. 2022). Indeed, pilot testing suggested these dimensions resulted in a reasonably realistic experience. The windows were not visible when participants looked forward toward a fixation dot, to avoid luminance influences on the pupil while participants were performing the task (Fig. 1). Participants were not embodied in the VE (i.e., they did not have a “virtual body” representing them), however the chair they were sitting on in the real world was present in the VE. Participants were seated 3 m from the back wall. Immediately in front of the participant was a desk and further behind that two chairs. The chairs were placed 4.5 m away from the participant, shifted 1m to the left or right and rotated to face the participant. A red fixation dot (diameter of 0.05 m) was placed in the middle of the wall that the participants were facing.

Virtual copresence was manipulated by adding two agents to the room during half of the trials. They were seated on the chairs facing the participant (Fig. 1A). Graphical representations of the agents (characterized as one female and one male) were taken from Adobe’s Mixamo (https://www.mixamo.com). The agents had individual looping idle animations representing breathing and small movements to make them more lifelike. The agents were able to provide evaluative feedback by either nodding (positive) or shaking (negative) their head, depending on the participant’s performance on the listening task. This feedback was given immediately after the experimenter had scored the participant’s response (see later). Agents were programmed so that each had a 15% chance of displaying feedback during any single trial (each condition contained 30 trials). However, in one test condition participants were unlikely to make errors (single-sentence condition; further described later). Therefore, the chance of displaying negative feedback (head shake) following an incorrect response was programmed to be 80% for that condition. In the single-sentence condition, positive feedback could be displayed whenever the full sentence was repeated correctly. In the dual-sentence condition, this only occurred when at least two words were correctly repeated from both sentences. In all other instances, there was a chance of receiving negative feedback. To ensure roughly the same amount of feedback between conditions, each agent could individually show a maximum of three negative and five positive feedback animations per condition. Specifically, during the easier conditions (single sentence; see later), either one or both of the agents gave negative feedback on an average of 3.0 (range 0 to 6) trials and positive feedback on an average of 8.0 (range 5 to 10) trials. For the harder conditions (dual sentence) there were an average of 3.7 (range 0 to 6) and 6.9 (range 1 to 9) occurrences, respectively.

During the conditions in which the agents were absent, visual distractors were shown in place of the agents. These consisted of a pile of cubes, arranged roughly in a humanoid shape and colored similarly to the agents (Fig. 1B). This controlled for the potential effect of the mere presence/absence of visual components in the VE on the pupil response. The cubes randomly wiggled in the *x*, *y*, and *z* directions (max: 0.01 cm) to mimic the idle animations of the agents. The cubes did not provide evaluative feedback.

### Task, Stimuli, and Outcome Measures

#### SiN Task

Participants performed a SiN task for which they listened to and repeated spoken Dutch sentences, presented through on-ear headphones attached to an HTC Vive Pro Eye HMD. The sentences, which were taken from Versfeld et al. (2000), varied in duration between 1.2 and 2.0 sec and contained on average six words (range: 4 to 9 words). On each trial, participants were presented with two sentences: one to the left ear (female voice) and one to the right ear (male voice). The sentences were masked by a single continuous noise masker with a similar long-term average frequency spectrum as the target speech, which was played to both ears simultaneously (duplicate segments) (Versfeld et al. 2000). For pupillometry purposes (Winn et al. 2018), the masker started playing three seconds before target sentence onset (which allowed for the pupil to react to the onset of the masker) and lasted for another three seconds after the offset of the longest of the two sentences (which allowed for the full pupil response to be captured). All sentences were played at −3 dB SNR with the masker calibrated to play at 65 dB SPL. This SNR was used as Koelewijn et al. (2014) observed the largest effects of task load on the PPD at this SNR.

The participant’s task was to either repeat only the sentence presented to the left ear (single-sentence condition) or repeat both sentences (dual-sentence condition), starting with the one presented to the left ear. Participants were instructed to delay their response until the masker sound had ended. After their response, an experimenter scored the words that were correctly repeated. Each condition consisted of 30 trials. Lists of sentences were precompiled and their assignment to conditions was counterbalanced between participants.

#### Pupillometry

During the task, participants’ pupil size was measured using the built-in eye tracker of the HTC Vive Pro Eye. Data of both eyes were recorded at roughly 90 Hz. The sampling frequency somewhat varied as it was coupled to the rate at which the experiment updated its frames (i.e., the speed at which calculations were performed to determine what should be displayed in the next frame). At the onset of every trial (start of the masker) a trigger was added to the data to separate data belonging to individual trials (pupil traces). This was done for the right eye only, which is thought to be more sensitive to cognitive effort than the left eye (Liu et al. 2017; Wahn et al. 2017). During recording, the eye-tracking system automatically marked samples taken while the eyes were closed, signaling blinks. Any trace with more than 20% marked values was removed and excluded from further analyses. If six traces or more within a condition were excluded, it was assumed that not enough data were available to properly approximate the pupil response, and that condition was removed from further analysis. All remaining traces were de-blinked. Because the opening and closing of the eye causes artifacts in the pupil data, all samples 83 msec before and 133 msec after marked segments were considered to be part of the blink (Siegle et al. 2008). Traces were resampled to 60 Hz and segments containing blinks were replaced through linear interpolation. Next, BPS was calculated for each trace by taking the average pupil size during the last second of noise before target sentence onset. Traces were baseline corrected by subtracting BPS from each sample in the trace. Last, all traces were smoothed using an 11-point moving average filter to remove high-frequency noise.

The first four traces of each condition were removed because there are some indications that pupil measures are unstable during these trials, as the participant is still adjusting to the task (Zekveld et al. 2018). We analyzed the data in the interval between masker onset and the length of the shortest trial (7.2 sec total; the shortest sentence had a duration of 1.2 sec). The data of trials within a condition were averaged to create one baseline corrected mean pupil trace per condition. Similarly, the BPS values of each trial within a condition were averaged, resulting in one mean BPS value per condition. Last, PPD was defined as the maximum value within the mean trace.

#### Subjective Ratings and Questionnaires

After each condition, participants were asked to rate task difficulty, effort, performance, and engagement. Responses were given on a visual analog scale using pen and paper while participants did not wear the HMD. The scale ranged from 1 to 10 and answers could be given with one decimal precision. At the end of the experiment, participants completed the Dutch Igroup Presence Questionnaire (Schubert et al. 2001), which had participants rate 14 items related to experienced PI using a five-point scale. All ratings from the IPQ were summed into a single score, representing the degree to which PI was experienced. Furthermore, participants were asked to rate the following five statements related to the agents using a seven-point scale (translated from Dutch):

- 1: I could sense the presence of the agents (strongly disagree – strongly agree).
- 2: It felt as if the agents were real (strongly disagree – strongly agree).
- 3: Because of the presence of the agents I felt … (less relaxed – more relaxed).
- 4: Because of the presence of the agents I felt … (encouraged – discouraged).
- 5: Compared to when the agents were absent, I tried to (do my best less – do my best more) when they were present.

The statements were specifically developed for the present experiment. It was assumed that for the agents to have a copresence effect on participants, the participants should regard them as somewhat real and that the participants were mindful of their presence. We aimed to assess the degree to which participants experienced the agents as copresent social entities by averaging items 1 and 2. Furthermore, it was assumed that participants might differ in their experience of the agents as something positive or negative, which could influence pupil and/or performance measures. To assess this, scores 4 to 5 were averaged into one score as well. Note that the scoring of item 4 was reversed so that higher numbers represented more perceived encouragement from the agents. Participants were asked to provide short written explanations of their ratings to provide the experimenters with additional insights.

### Procedure

After signing informed consent, participants underwent testing to confirm that they met the audiometric criteria. They were then fitted with the HTC Vive Pro Eye HMD. Before starting the SiN testing, participants were presented with four practice trials: two in the single-sentence condition and two in the dual-sentence condition. During practice, participants were shown the visual distractors, agents and all possible animations to familiarize them with the test environment. Participants were instructed to fixate on the red fixation dot in front of them whenever sound was playing. Following the practice trials, participants completed four experimental conditions, the order of which was counterbalanced across participants. Before each experimental condition, the eye-tracker was calibrated and participants were shown a 3-min clip of aerial shots from Scotland for baseline cardiovascular measurements (described elsewhere; Plain et al. in prep). Between each test condition the HMD was removed so participants could complete the subjective ratings. Participants were then offered a short break of a couple of minutes, after which they resumed the experiment. At the end of the experiment, participants completed the PI questionnaire and the agent-related ratings.

### Data Analysis

Performance was scored as the percentage of words repeated correctly. In line with Koelewijn et al. (2014), this percentage was calculated across one sentence in the single-sentence condition and across both sentences in the dual-sentence condition. However, performance scores in the single-sentence conditions were often near or at ceiling, making it impossible to analyze them using frequentist statistics. Therefore, the dual-sentence distractor and agent conditions were compared using paired-samples *t*-test.

BPS and PPD were modelled as dependent variables using linear mixed effect models, created with the lme4 (Bates et al. 2015) package in R programming language (R Core Team 2020). The models included task load, virtual copresence, and their interaction as fixed effects, together with the random intercept. Random slopes were not included to avoid over-fitting and convergence problems of the models. Parameter estimates were extracted from the models and *F*-statistics were calculated using Satterthwaite’s (Satterthwaite 1946) degrees of freedom.

As an exploratory analysis, trials were separated into conditions based on whether the agents provided positive, negative, or no feedback in the prior trial. Traces belonging to trials following positive feedback were averaged into one mean trace and the same was done for traces following no feedback. The trials following negative feedback were ignored as these occurred too infrequently to properly capture the pupil dilation response within the remaining trials. The positive feedback condition was excluded if there were fewer than six traces with a recording of sufficient quality (four occurrences in total). A model including fixed effects for task load, feedback (positive or none during the previous trial) and their interaction was fitted to the BPS and PPD data.

As an exploratory analysis, the IPQ scores measuring degree to which participants experienced the agents as copresent social entities, and the scores representing the degree to which participants experienced the agents as copresent social entities and as something positive or negative were added to the models predicting PPD and BPS. These models were then compared with the original models using a log-likelihood test.

Subjective rating data were analyzed in the same manner as the pupil data, excluding the exploratory analyses. Furthermore, it was assessed if the subjective ratings correlated (Pearson’s correlation) with pupil size measures.

## RESULTS

### Exclusions

On several occasions, pupil data quality was not sufficient and all data from a condition had to be excluded. This resulted in the exclusion of one full participant (whose pupils were difficult to properly measure), one “single sentence alone” condition and one “dual sentence alone” condition. Data of participants with missing conditions were included in the analyses where possible.

### Performance

**Fig. 2.**
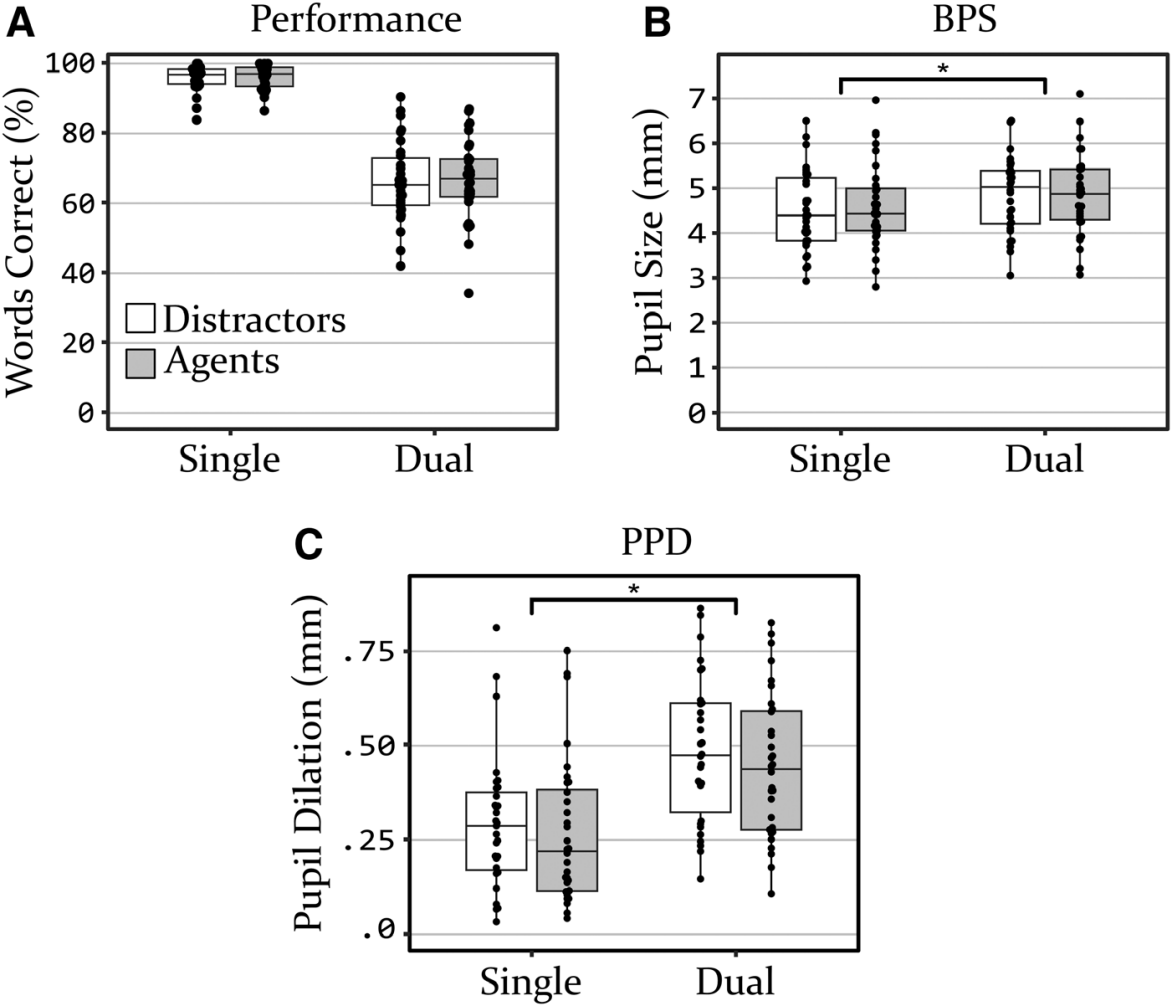
Plots of (A) the percentage of words repeated correctly, (B) BPS, and (C) PPD. Asterisks correspond to significant effects as found by the *F*-test. BPS indicates baseline pupil size; PPD, peak pupil dilation.

Mean word repetition performance has been plotted in Figure 2A. Performance on the dual-sentence task condition was associated with a smaller overall percentage of correctly repeated words. However, as noted earlier, this was not tested using statistics. For most participants, performance was better for the sentence presented to the left ear (80.0% words correct on average) than for the sentence presented to the right ear (53.0% words correct on average); *t*(61) = 10.0, *p* < 0.01. This was probably because participants were instructed to prioritize the left ear sentence. A paired-samples *t*-test comparing performance between the virtual copresence conditions in the dual-sentence condition indicated no significant difference, *t*(29) = −0.30, *p* = 0.77.

### Baseline Pupil Size

The mean BPS is plotted in Figure 2B. Parameter estimates of the linear mixed effect model and their CIs can be found in Table 1. The *F*-test revealed a significant main effect of task load, *F*(1,93) = 20.24, *p* < 0.01. As indicated by the positive parameter estimate, BPS was significantly higher during dual-sentence conditions, compared with single-sentence conditions. However, the CIs suggest that there was considerable variation between participants. No significant main effect was found for virtual copresence, *F*(1,93) = 0.27, *p* = 0.60, nor was there a significant interaction between task load and virtual copresence, *F*(1,93) = 0.38, *p* = 0.54.

**TABLE 1.** Parameter estimates and 95% CIs of BPS and PPD

	BPS			PPD		
	Estimate	CI Lower	CI Upper	Estimate	CI Lower	CI Upper
Intercept	4.52	4.22	4.83	0.29	0.23	0.36
Load	**0.32**	**0.14**	**0.49**	**0.18**	**0.13**	**0.24**
Copresence	0.07	−0.10	0.24	−0.02	−0.07	0.03
Load × Copresence	−0.08	−0.32	0.17	−0.01	−0.08	0.07

Parameter estimates for load represent the change from single-sentence load to dual-sentence load. The estimate for copresence represents the change from copresent distractors to copresent agents. Rows in bold correspond to significant effects as found using *F*-tests.

BPS, baseline pupil size; CI, confidence interval; PPD, peak pupil dilation.

### Peak Pupil Dilation

**Fig. 3.**
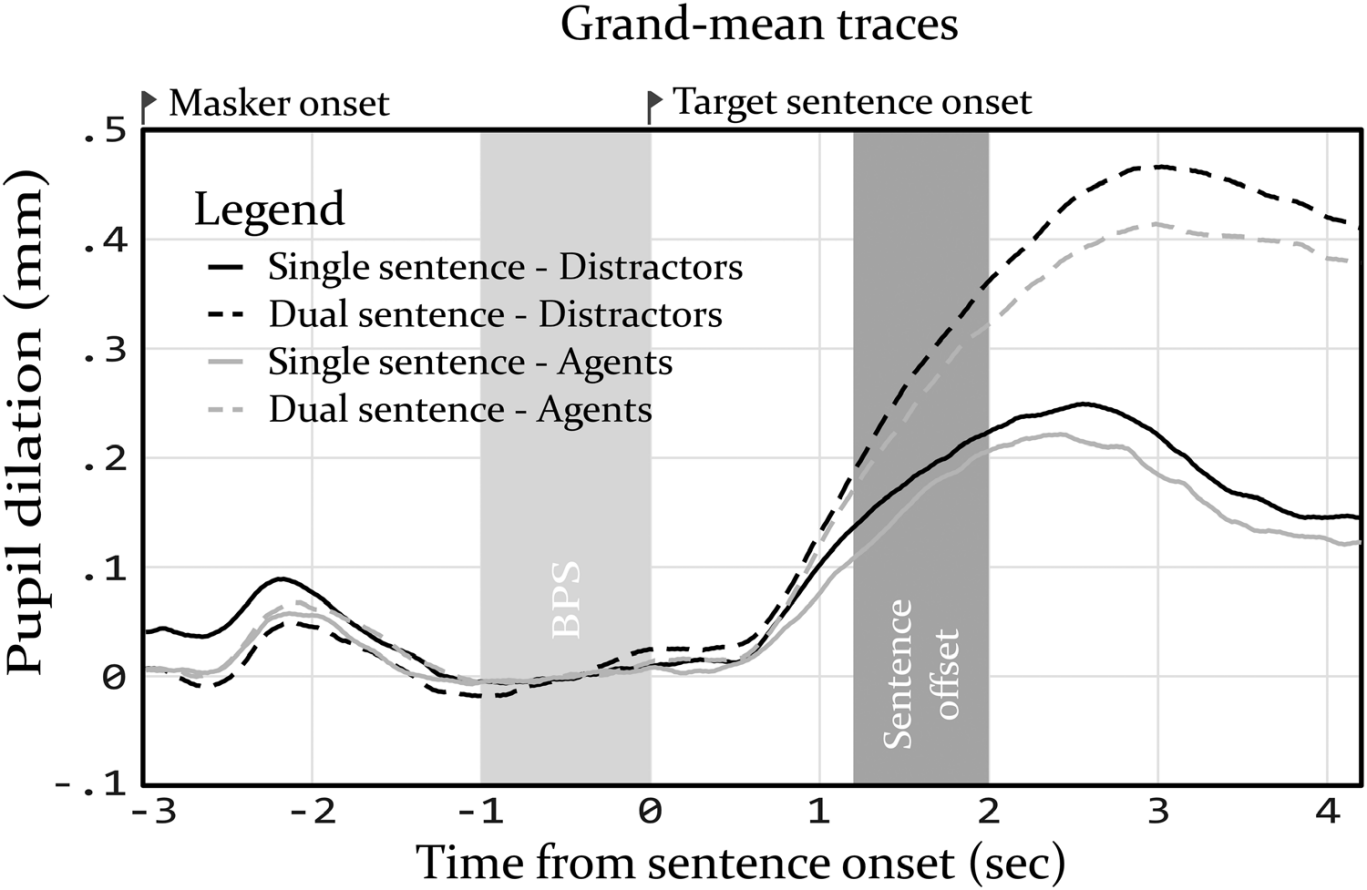
Plots of average pupil traces of each condition. At −3 sec, the masker started playing. Zero seconds marks the onset of the target sentence. The gray box between 1.2 and 2.0 sec marks the period during which target sentences stopped playing. BPS indicates baseline pupil size.

Traces of the full pupil response (averaged over participants) have been plotted in Figure 3. The overall morphology of the pupil dilation responses is similar to that commonly found in speech perception research using pupillometry (Koelewijn et al. 2014; Winn et al. 2018; Pielage et al. 2021), with a clear peak 2.5 to 3 sec after target sentence onset. A trend is visible whereby the dual-task conditions resulted in a greater amplitude of the pupil dilation response. Furthermore, another trend is visible whereby the presence of the agents seems to be associated with a slightly reduced amplitude of the pupil dilation response, compared with conditions where they were absent.

The mean PPD in the four conditions is plotted in Figure 2C. Model parameter estimates and CIs can be found in Table 1. The *F*-test revealed a significant effect of task load on PPD, *F*(1,93) = 93.99, *p* < 0.01. The associated parameter estimate for task load confirmed that PPD increased for dual-sentence conditions, compared with single-sentence conditions. The *F*-test did not reveal a main effect of virtual copresence on PPD, *F*(1,93) = 1.91, *p* = 0.17, nor an interaction between task load and virtual copresence, *F*(1,93) = 0.03, *p* = 0.87.

### Feedback

Parameter estimates can be found in Table 2. The *F*-test revealed a significant main effect for task load, *F*(1,92) = 19.37, *p* < 0.01, and feedback, *F*(1,92) = 5.29, *p* = 0.02, on BPS. The associated parameter estimate suggests that BPS was higher for trials following positive feedback, compared with no feedback. It should be noted that the confidence intervals were rather wide, suggesting much variability between participants. Furthermore, the parameter’s confidence intervals encompass zero, meaning this effect must be interpreted with care. There was no significant interaction between task load and feedback, *F*(1,92) = 0.00, *p* = 0.96.

**TABLE 2.** Parameter estimates and 95% CIs of BPS and PPD when modeling task load and feedback

	BPS			PPD		
	Estimate	CI Lower	CI Upper	Estimate	CI Lower	CI Upper
Intercept	4.05	3.77	4.33	0.28	0.21	0.35
Load	**0.19**	**0.07**	**0.30**	**0.18**	**0.12**	**0.24**
Feedback	**0.10**	**−0.01**	**0.21**	0.02	−0.04	0.08
Load × Feedback	−0.01	−0.17	0.16	−0.04	−0.12	0.05

Parameter estimates for load represent the change from single-sentence load to dual-sentence load. The estimate for feedback represents the change from no feedback on the previous trial to positive feedback on the previous trial. Rows in bold correspond to significant effects as found using *F*-tests.

BPS, baseline pupil size; CI, confidence interval; PPD, peak pupil dilation.

Similar to the data of BPS, a model including fixed effects for task load, feedback (positive or none on previous trial) and their interaction was fitted to the PPD data. The *F*-test found the expected main effect of task load, *F*(1,93) = 57.37, *p* < 0.01, but found no effect of feedback, *F*(1,93) = 0.01, *p* = 0.92, nor an interaction between task load and feedback, *F*(1,93) = 0.72, *p* = 0.40. Parameter estimates can be found in Table 2.

### Questionnaires

The mean IPQ score was 41.1 (SD = 8.4). The grand mean score of agent-related ratings items 1 and 2 was 4.3 (SD = 1.1). For items 3 to 5, the mean was 3.9 (SD = 0.9). Neither the IPQ score, nor the scores of the agent questionnaire significantly contributed to the amount of variance explained by the models (Table 3).

**TABLE 3.** Results of log-likelihood tests when adding questionnaire scores to the models predicting BPS and PPD

Fixed Factor	BPS			PPD		
	df	Χ^2^	*p*	df	Χ^2^	*p*
Igroup Presence Questionnaire score	4	1.11	0.89	4	4.16	0.38
Mean of items 1–2 on the agents questionnaire	4	7.44	0.11	4	1.75	0.78
Mean of items 3–5 on the agents questionnaire	4	6.79	0.15	4	5.29	0.26

BPS, baseline pupil size; df, degrees of freedom; PPD, peak pupil dilation.

### Subjective Ratings

Results of the *F*-test and parameter estimates can be found in Table 4. All subjective ratings, except for “engagement” showed a significant main effect for task load. This indicated higher effort, lower performance and higher difficulty ratings for the dual-sentence compared with the single-sentence conditions. No main effect of virtual copresence or interaction was found. Significant weak to moderate correlations were found between the effort, performance and difficulty subjective ratings and PPD (Table 5). The engagement rating did not correlate with PPD and none of the ratings correlated with BPS.

**TABLE 4.** Parameter estimates and 95% CIs of the subjective ratings

	Effort					
	df	*F*	*p*	Estimate	CI Lower	CI Upper
Intercept				5.13	4.58	5.69
Load	**1, 95**	**230.94**	**<0.01**	**2.88**	**2.33**	**3.42**
Copresence	1, 95	0.04	0.84	−0.13	−0.66	0.40
Load × Copresence	1, 95	0.21	0.65	0.18	−0.58	0.94
	Performance					
	df	*F*	*p*	Estimate	CI Lower	CI Upper
Intercept				8.50	8.06	8.93
Load	**1, 96**	**453.90**	**<0.01**	**−3.49**	**−3.92**	**−3.07**
Copresence	1, 95	0.00	0.98	−0.26	−0.67	0.16
Load × Copresence	1, 95	2.91	0.09	0.52	−0.08	1.11
	Engagement					
	df	*F*	*p*	Estimate	CI Lower	CI Upper
Intercept				6.83	6.02	7.64
Load	1, 96	0.38	0.54	0.36	−0.15	0.88
Copresence	1, 96	1.18	0.28	0.45	−0.06	0.95
Load × Copresence	1, 96	1.81	0.18	−0.50	−1.22	0.23
	Difficulty					
	df	*F*	*p*	Estimate	CI Lower	CI Upper
Intercept				3.95	3.38	4.53
Load	**1, 94**	**351.78**	**<0.01**	**3.93**	**3.36**	**4.49**
Copresence	1, 93	0.16	0.69	0.18	−0.37	0.74
Load × Copresence	1, 93	0.25	0.62	−0.20	−1.00	0.59

Parameter estimates for load represent the change from single-sentence load to dual sentence load. The estimate for copresence represents the change from copresent distractors to copresent agents. Rows in bold correspond to significant effects as found using *F*-tests.

CI, confidence interval; df, degrees of freedom.

**TABLE 5.** Pearson’s correlations between subjective ratings and PPD (df = 121)

	PPD			BPS		
	*r*	*t*	*p*	*r*	*t*	*p*
Effort	**0.27**	**2.03**	**<0.01**	0.01	0.10	0.91
Performance	**0.32**	**3.67**	**<0.01**	−0.08	−0.89	0.37
Engagement	−0.12	−1.35	0.18	−0.02	−0.22	0.83
Difficulty	**0.32**	**3.67**	**<0.01**	0.09	0.95	0.35

Rows in bold correspond to significant correlations. df, degrees of freedom; PPD, peak pupil dilation.

## DISCUSSION

This study assessed if pupil measures taken from within an HMD were sensitive to a task load manipulation during a speech perception task. It also assessed whether the copresence of agents (computer-steered renderings of persons) affected performance, measures of pupil size and/or subjective ratings. Participants were simultaneously presented with two sources of masked target speech and were asked to either attend only one source, or to attend both. They did this either while they were accompanied by two copresent agents or while they were accompanied by non-agent distractors in the VE.

The manipulation of task load (attending one or two sources) significantly increased PPD as well as BPS. The virtual copresence manipulation (copresent agents or distractors) did not affect task performance or pupil metrics. However, there was some evidence that receiving positive feedback from the agents was associated with increased BPS on the following trials. Subjective scores associated with the experience of PI and the perception of the agents were not related to the pupil data. Besides task engagement, all other subjective ratings indicated that the dual-sentence condition was more difficult, effortful, and resulted in lower estimated performance than the single-sentence condition. However, none of the subjective ratings were influenced by virtual copresence condition.

Our finding that PPD, as extracted from pupil measurements within an HMD, was sensitive to the manipulation of task load (attending either one or two sentences) is in line with the data of Koelewijn et al. (2014). When comparing the pupil data as shown in Figure 3 and Table 1 to those of Koelewijn et al., a similar magnitude can be found in the effect of task load on PPD. In both studies, attending two sentences simultaneously seemed to elicit a pupil dilation response with a peak of approximately 0.4 mm, which was approximately 0.2 mm greater than attending a single sentence. The results follow the commonly found pattern that increasing listening task load results in greater PPDs (Zekveld et al. 2018), which is commonly associated with increased effort. Indeed, subjective ratings of effort, performance and difficulty correlated significantly with PPD, suggesting that PPD increased when the task was perceived as more difficult and effortful. By replicating these findings using a VR-HMD, we obtained evidence that listening effort can be studied using a combination of pupillometry and VR-HMDs.

Manipulating virtual copresence via VR did not affect PPD or BPS pupil measures. This contrasts with studies that manipulated virtual copresence by means of physical persons (Pielage et al. 2021, 2023). A possible explanation is that the VE did not elicit sufficient degrees of PI for participants to react to it as if it were real (Slater & Sanchez-Vives 2016). Indeed, we found a mean IPQ score of 41.1 (possible range: 14 to 70), which is somewhat lower than the scores of 50 and above found in previous studies using HMDs (Krijn et al. 2004; Vasconcelos-Raposo et al. 2016; Narciso et al. 2019). Furthermore, when asked if they felt that the agents were real, participants generally provided a relatively low score (2.8 on a scale of 1 to 7). More sophisticated and realistic VEs would likely have elicited larger effects of virtual copresence (Zibrek & McDonnell 2019; Li et al. 2020).

BPS was measured while participants were anticipating the onset of the target sentence(s) (Ayasse & Wingfield 2020). Our finding that BPS was higher for dual-sentence conditions as compared with single-sentence conditions suggests that higher anticipated task load may have caused pre-mobilization of effort or higher levels of arousal.

An exploratory analysis, we found that BPS increased on trials that followed positive feedback by the agents, compared with trials where no feedback was given. It could be that the change in BPS reflects participants adjusting their anticipated task load or arousal following positive feedback. However, there is uncertainty about calculating *p* values (or specifically, the degrees of freedom) with linear mixed-effect models (Luke 2017). Because the confidence intervals of the estimated parameter contain zero, it can be questioned if the statistical significance of the effect has any practical relevance. Regardless, the findings signal that feedback may be able to influence pupil size measures like BPS. Or, because positive feedback was associated with increased BPS on the next trials and feedback was tied to performance, the results may also signal a relationship between performance and BPS on the next trial. Because feedback is part of real-life listening, it is important to better understand how it affects the pupil. While this study was not designed with with feedback analyses in mind, future research could contribute to better understanding of the role of feedback in (copresence) listening research. Such studies should include a manipulation of feedback (positive and negative) in a sufficient number of trials and use an appropriate control condition.

The finding that subjective effort, performance, and difficulty were sensitive to task load is consistent with other data (Zekveld et al. 2010; Koelewijn et al. 2014, 2021; Wu et al. 2016). It confirms that participants experienced increased task load when having to attend two sources of speech, compared with one. Virtual copresence did not affect the subjective ratings, suggesting that the virtual copresence of the agents did not affect the experienced performance, effort, engagement or the task’s difficulty. This is in line with the agent questionnaire scores, which showed that participants generally felt indifferent toward the agents. Participants often noted that the agents: “were clearly not real people” and “static.” The participants who did report being somewhat affected by the agents, said they felt more “encouraged to perform well, to avoid disapproval” and that “the nodding [of the agents] was a little encouraging.”

A limitation was that this study used a simple VE that looked like a laboratory. While this was useful to replicate findings commonly acquired in the laboratory, no conclusions can be made regarding its application to more sophisticated VEs. For example, in this study, participants fixated on a single point in virtual space, while a more ecologically valid VE would have participants look around the space. Head movements will change luminance within the HMD display, likely affecting pupil size. The impact of this needs further exploration. Another limitation of this study was that a strong task load manipulation was used. Although this was done to increase the likelihood of being able to measure an effect on pupil size, situations where two sources of speech must be attended simultaneously are rare in daily life. It is thus important that less extreme task load manipulations be examined in future studies.

Further recommendations for future studies include assessing if more advanced VEs (e.g., by means of photorealistic graphics and motion captured agent animations) are able to influence participant performance, pupil measures (and/or other physiological measurements) and subjective ratings. Possibly, linking the sounds to the visual characteristics (e.g., using a masker that seemingly originates from a visible sound source in the environments, such as a noisy machine or people talking in the background) would also result in a more realistic environments. Furthermore, it would be useful to gain additional insights into the effects of negative and positive feedback on pupil measures during speech perception tasks. Studies designed for that purpose could also assess if the origin of the feedback (e.g., by a person or by a computer) matters.

The results are promising with respect to the application of VR in experimental and, eventually, even clinical studies. Although the effects of hearing loss on the pupil response (in VR) require more research, there is no reason to assume that the successful application of VR in speech perception test paradigms is influenced by hearing loss in itself. As such, these results may pave the way for VR tests that are able to assess hearing (loss) in highly ecological environments.

## CONCLUSION

Pupillometry in VR-HMDs was found to be sensitive to manipulations of auditory task load. VR can thus be successfully combined with pupillometry in speech perception research. As opposed to earlier non-VR studies, the virtual copresence manipulation used in this study did not affect participants’ performance, pupil size measures, or subjective ratings. Noninteractive and relatively simple social VEs might thus not be sufficient to research the effects of copresence on speech perception. More sophisticated and technologically advanced virtual testing environments might be required. Last, it seems that receiving feedback on speech perception performance might affect how much effort is mobilized in anticipation of the next trial. However, it is not clear if this is only the case for feedback from an agent and if it differs between positive and negative feedback.

## ACKNOWLEDGMENTS

This project has received funding from the European Union’s Horizon 2020 research and innovation program under the Marie-Sklodowska-Curie grant agreement No. 765329. G.H.S. received support from the NIHR Manchester Biomedical Research Centre.
